# Low-intensity transcranial ultrasound stimulation facilitates hand motor function and cortical excitability: A crossover, randomized, double blind study

**DOI:** 10.3389/fneur.2022.926027

**Published:** 2022-09-05

**Authors:** Meng-Fei Zhang, Wei-Zhou Chen, Fub-Biao Huang, Zhi-Yong Peng, Ying-Chan Quan, Zhi-Ming Tang

**Affiliations:** ^1^Department of Rehabilitation Medicine, Yuedong Hospital, The Third Affiliated Hospital of Sun Yat-sen University, Meizhou, China; ^2^Department of Occupational Therapy, China Rehabilitation Research Center, Beijing, China; ^3^Department of Rehabilitation Medicine, The Third Affiliated Hospital of Sun Yat-Sen University, Guangzhou, China

**Keywords:** transcranial ultrasound, hand function, neuromodulation, motor evoked potential, motor cortex

## Abstract

**Objective:**

Transcranial ultrasound stimulation (TUS) is a new form of non-invasive brain stimulation. Low-intensity TUS is considered highly safe. We aimed to investigate the effect of low-intensity TUS on hand reaction responses and cortical excitability in healthy adults.

**Methods:**

This study used a crossover, randomized, and double-blind design. A total of 20 healthy participants were recruited for the study. All the participants received TUS and sham stimulation on separate days in random order. The finger tapping test (tapping score by using a tablet) and motor evoked potential (MEP) were assessed before and after stimulation, and discomfort levels were assessed using a visual analog scale (VAS) score.

**Results:**

No significant differences in tapping score or MEP amplitude between the two experimental conditions were registered before stimulation. After stimulation, tapping scores were increased regardless of the specific treatment, and the real stimulation condition receiving TUS (90.4 ± 11.0 points) outperformed the sham stimulation condition (86.1 ± 8.4 points) (*p* = 0.002). The MEP latency of real TUS (21.85 ± 1.33 ms) was shorter than that of sham TUS (22.42 ± 1.43 ms) (*p* < 0.001). MEP amplitude of real TUS (132.18 ± 23.28 μV) was higher than that of sham TUS (114.74 ± 25.5 μV, *p* = 0.005). There was no significant difference in the discomfort score between the two conditions (*p* = 0.163).

**Conclusion:**

Transcranial ultrasound stimulation (TUS) can decrease the hand reaction response time and latency of the MEP, enhance the excitability of the motor cortex, and improve hand motor function in healthy individuals without obvious discomfort.

## Introduction

Neuromodulation techniques, which can change central excitability and induce neuroplasticity, have been successfully used for rehabilitation after central neural system injury or function disorder (1, 2). Transcranial magnetic stimulation (TMS) and transcranial direct current stimulation (tDCS) have been widely studied in the past (3, 4). Transcranial Ultrasound stimulation (TUS), which can pass through the skull and influence central nervous function mechanically, has become a research focus for research in recent years (5). As a novel neuromodulation technique, TUS has the advantages of being non-invasive and providing high spatial resolution and greater penetration depth. Preliminary studies suggest that TUS can locally regulate sensory induction and cortical function (6).

Ultrasound can be classified into high, medium, or low intensities according to the power applied. High-intensity ultrasound (>200 W/cm^2^) mainly inhibits neuronal activity through nerve ablation, while medium-intensity ultrasound (100–200 W/cm^2^) is normally used to disrupt the blood-brain barrier non-invasively. Low-intensity ultrasound (<100 W/cm^2^), on the other hand, does not involve a significant accumulation of thermal energy, which may cause DNA fragmentation, coagulative necrosis, and cell death (7, 8), relying instead on a direct mechanical effect (9).

Previous studies mostly focused on animal experiments-very few studies discussed the effect of TUS on the human cortex (10, 11). The animal studies reported both excitation and inhibition effects of low-intensity TUS on the central neural system (12, 13). One human study reported intervention in the right prefrontal gyrus in healthy individuals and found an inhibitory effect on the cortex but a positive effect on mood (14). The therapeutic effect of TUS on the cortex may be related to parameters, such as focused or non-focused, intensity, frequency, and stimulation location (15, 16). Focused TUS and non-focused TUS can lead to a differential stimulation effect according to the volume of the brain tissue impacted (17). Frequency and intensity may cause different physiological effects (8). In humans, TUS delivered to the M1 region at 0.50 MHz, 6.16 W/cm^2^ inhibited neuronal excitability (11). Diagnostic imaging ultrasound (unfocused TUS) stimulation with a frequency of 2.32 MHz and intensity of 34.96 W/cm^2^ increased neuronal excitability (10). Using multiple Focused TUS transducers to stimulate the primary sensory cortex elicited various tactile sensations in the absence of any external sensory stimuli (18). Sanguinetti reported a focused TUS study with a randomized, placebo-controlled, double-blind design, in which participants received 30 s of 500 kHz focused TUS or a placebo control. They found that focused TUS can be used to modulate mood and emotional regulation networks in the prefrontal cortex when targeting the right inferior frontal gyrus in healthy human volunteers (14).

In this study, we used the tapping action and motor evoked potentials (MEPs) (19) to investigate the effect of low-intensity TUS on hand motor function and cortical excitability in healthy adults.

## Materials and methods

### Participants

According to the data collected from our preliminary experiment, a total of 20 healthy individuals were recruited for the study voluntarily (Table 1) [mean age (32.8 ± 13.4), the age range: 21–59, women: 12, men: 8]. The inclusion criteria were: (1) being between 20 and 60 years of age, and (2) being right-handed. The exclusion criteria were: (1) having poor cooperation due to cognitive or hearing impairment, and (2) having a history of hand trauma. All participants were informed about the study design and objectives and signed informed consent. The study was approved by the Ethics Committee of Yue Dong Hospital of the Third Affiliated Hospital of Sun Yat-sen University (project 8, 2021).

**Table 1:** General information on subjects and stimulation methods.

**Subjects**	**Age**	**Gender**	**Stimulation methods**	
			**First stimulus**	**Second stimulus**
1	20	M	Sham TUS	Real TUS
2	21	M	Real TUS	Sham TUS
3	21	M	Real TUS	Sham TUS
4	23	M	Sham TUS	Real TUS
5	22	W	Real TUS	Sham TUS
6	29	M	Real TUS	Sham TUS
7	43	W	Sham TUS	Real TUS
8	29	W	Real TUS	Sham TUS
9	22	M	Sham TUS	Real TUS
10	46	W	Real TUS	Sham TUS
11	32	M	Real TUS	Sham TUS
12	23	W	Sham TUS	Real TUS
13	51	W	Real TUS	ShamTUS
14	58	M	Sham TUS	Real TUS
15	25	W	Real TUS	Sham TUS
16	39	W	Real TUS	Sham TUS
17	59	W	Real TUS	Sham TUS
18	49	W	Sham TUS	Real TUS
19	23	W	Sham TUS	Real TUS
20	22	W	Real TUS	Sham TUS

M, Men; W, Women.

### Study design

**Figure 1:**
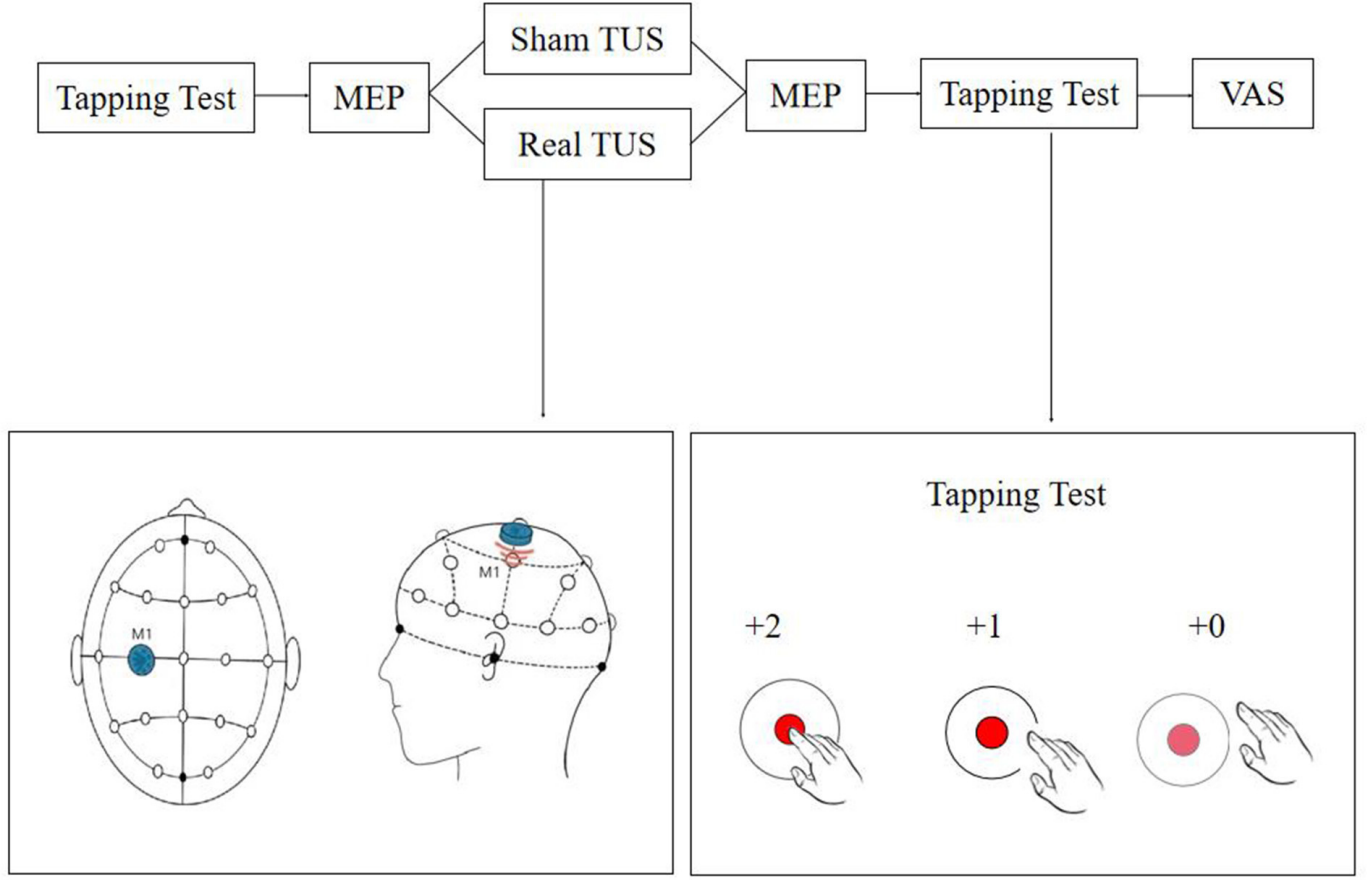
Flow diagram of the study. The tapping test and motor evoked potentials (MEPs) were recorded before and after stimulation, and discomfort levels were assessed using a visual analog scale (VAS) score.

A double-blind randomized crossover control design was used in this study. Random numbers were generated and recorded by a designated person who did not participate in any other operation or assessment. Each participant received real TUS and sham TUS with an interval of more than 24 h between each intervention to avoid potential residual effects, and the stimulation sequence was determined at random. The evaluator did not participate in the stimulation procedure (Figure 1).

### Experimental conditions

Transcranial ultrasound stimulation (TUS) stimulation (UE860A, Beijing Ruao Medical Technology Co., LTD., China) was provided once to each participant for a total duration of 10 min using a power of 1.2 W/cm^2^ and a frequency of 0.8 MHz, stimulation duration was 1 s and rest 2 s on-off ratio of 1:2 (20–22). The center of the probe (diameter = 20 mm) was placed on the “hotspot” determined by TMS as explained in the next section (2.4 evaluation). Participants were seated comfortably, and the surrounding environment was kept comfortable and quiet throughout the treatment session. For the sham stimulations, the ultrasound probe was placed in reverse. The TUS instrument was still in “turn on” state and all the other conditions were kept identical.

### Evaluation

The tapping score and MEP parameters of each participant were measured before and after the stimulation session, and the discomfort of the subjects during the stimulation was assessed. A tapping test based on the Quiq 2.0 APP software was used to quantify hand motor response (23). During the test, participants were required to tap on the moving point displayed on the screen of a tablet as quickly and as accurately as possible within 30 s. One point was awarded for each on-target tap and two points when the participant tapped in the inner circle. Three tests were performed before and after stimulation, and the average value was recorded in each case.

The TMS instrument and supporting MEP monitoring module (Wuhan Yiruide Medical Equipment New Technology Co., LTD., China) were used for measuring MEPs. The recording and reference electrodes were placed on the muscle belly and the tendon of the right abductor pollicis brevis, respectively, and the ground electrode was placed on the left wrist. The location for the stimulation was determined using the EEG 10-20 system, and the C3 position was marked on the head. Stimulation was provided around the C3 position range at 70% intensity using the 8-shaped coil. The coil was then moved until the best location to record the MEP was found (“hotspot”), the intensity was reduced, and the handle was positioned at a 45° angle to the middle line of the body. The resting motor threshold (rMT) was set at an intensity capable of inducing MEPs higher than 50 μV in at least five out of ten trials (19). The latency and amplitude of the hand MEPs before and after stimulation were recorded using a 120% rMT intensity. Ten independent measurements were recorded before and after stimulation, and the average value was considered for the analysis (24–26). The interval of each MEP measurement was 5–10 s.

A visual analog scale (VAS) was used to evaluate the degree of discomfort during the session, with zero considered as very comfortable and ten as extremely uncomfortable.

### Statistical analysis

We used G Power Statistics (3.1.9.2 version) to calculate the sample size of this study according to the results of pre-experiment. To achieve 80% power and 5% statistical significance, a sample size of 20 cases was required for this study. The SPSS 23.0 statistical software (IBM corporation, USA) was used for data analysis. Shapiro–Wilk test was used to confirm if the data were normally distributed. Levene statistic method to test if the variance was equal. Tapping score, MEP latency, and MEP amplitude were expressed as mean ± standard deviation. Two-factor repeated measurement ANOVA was used to test the main effect and interaction of conditions (real and sham) and time points (before and after stimulation). Paired *t*-test was used as a post pairwise comparison to determine differences between the two conditions. Also, paired *t*-test was used to determine the differences between before and after stimulation. VAS was presented as median (P25, P75), Wilcoxon signed ranks test was used to compare the difference between the two conditions. *p* < 0.05 was considered statistically significant.

## Results

### Tapping score

**Figure 2:**
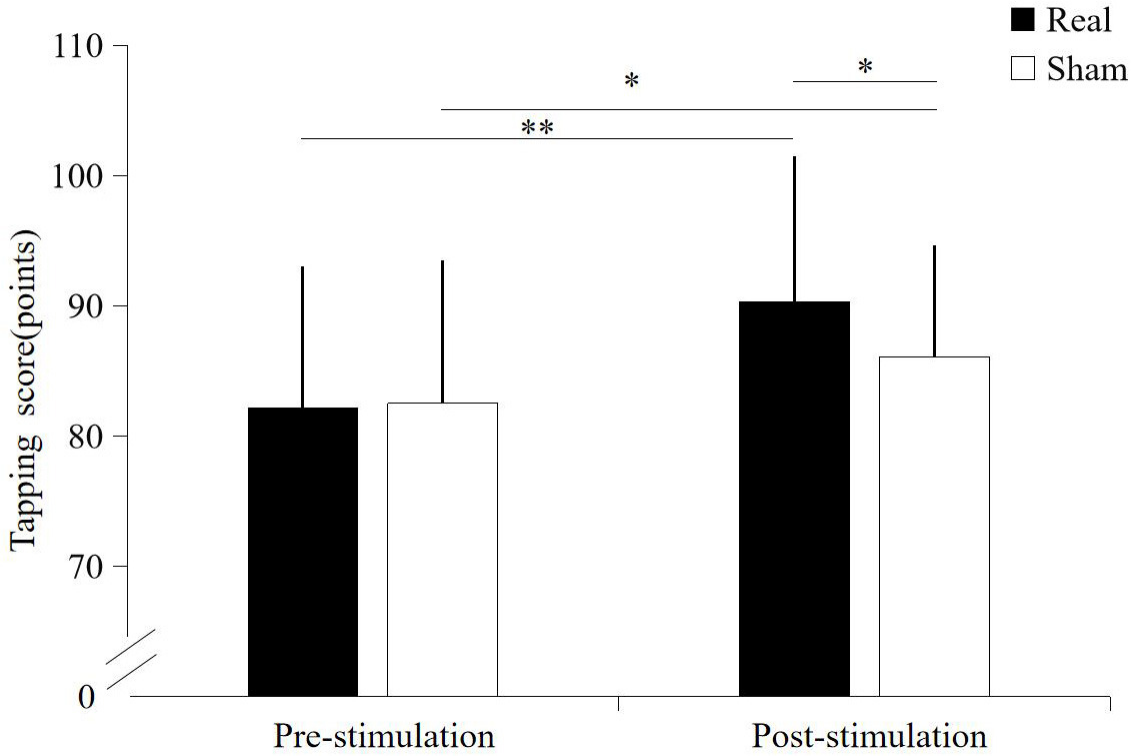
The results of the tapping score under both stimulation conditions. *p < 0.05, **p < 0.01.

A significant interaction effect was found between the condition and time point (*F* = 16.156, *p* < 0.001). Significantly main effect was found on condition (*F* = 8.120, *p* = 0.010) and time point (*F* = 59.323, *p* < 0.001). Before stimulation, there was no significant difference in tapping score between the real TUS condition (82.2 ± 10.75 points) and sham TUS conditions (82.5 ± 10.8 points, *t* = −0.758, *p* = 0.458). Moreover, the tapping score after real TUS (90.4 ± 11.02 points) was significantly higher than that obtained after the sham TUS (86.1 ± 8.4 points, *t* = 3.556, *p* = 0.002) (Figure 2). Tapping score became significantly higher after stimulation compared to before stimulation in both real TUS conditions (*t* = 11.2, *p* < 0.001) and sham TUS (*t* = 3.153, *p* = 0.005).

### Motor evoked potential latency

**Figure 3:**
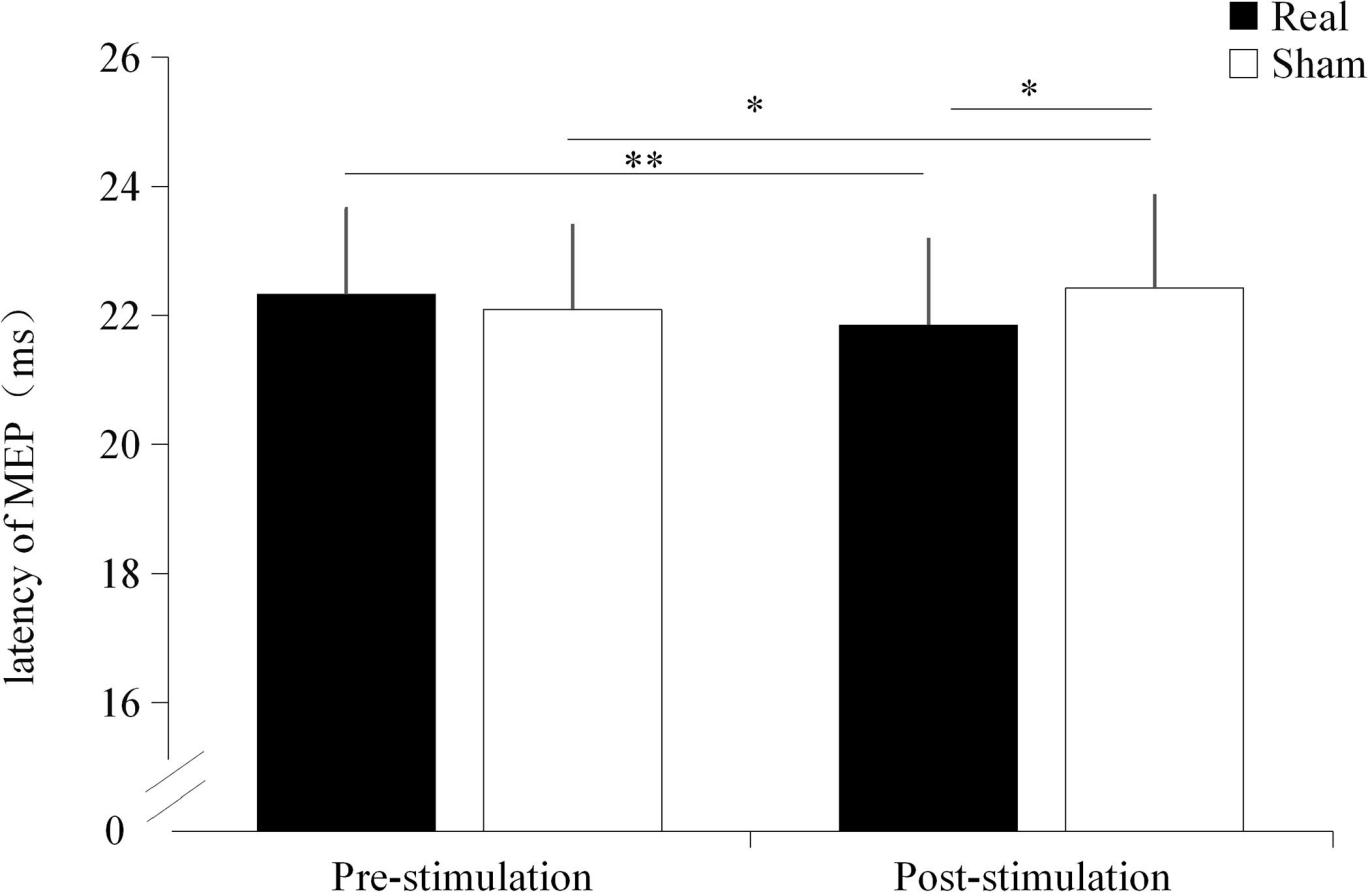
The latency of motor evoked potentials (MEPs) under both stimulation conditions. *p < 0.05, **p < 0.01.

A significant interaction effect was found between the condition and time point (*F* = 29.188, *p* < 0.001). No significantly main effect were found on condition (*F* = 1.314, *p* = 0.266) and time point (*F* = 0.869, *p* = 0.363). There was no significant difference in MEP latency before the stimulation between the real TUS condition (22.33 ± 1.32 ms) and sham TUS conditions (22.09 ± 1.30 ms, *t* = 1.295, *p* = 0.211). Moreover, the MEP latency after real TUS (21.85 ± 1.33 ms) was significantly shorter than those obtained after the sham TUS (22.42 ± 1.43 ms, *t* = −4.119, *p* < 0.001, Figure 3). MEPs became significantly shorter after stimulation compared to before stimulation in real TUS conditions (*t* = −7.889, *p* < 0.001), however they were longer under the sham TUS (*t* = 2.184, *p* = 0.042, Figure 3).

### Motor evoked potential amplitude

**Figure 4:**
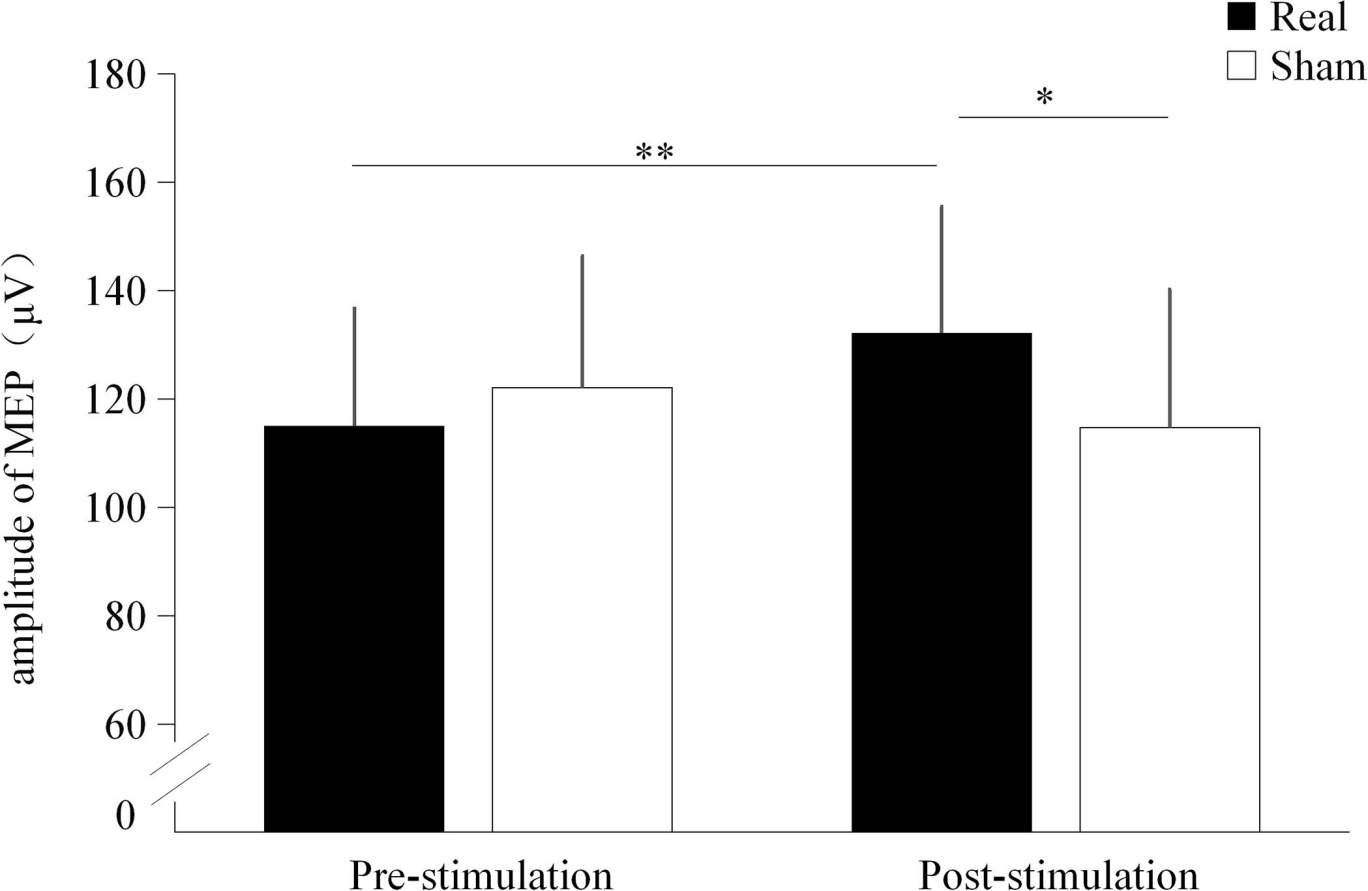
The amplitude of the motor evoked potentials (MEPs) under both stimulation conditions. *p < 0.05, **p < 0.01.

Significant interaction effect was found between the condition and time point (*F* = 15.822, *p* < 0.001). No significant main effect were found on condition (*F* = 2.312, *p* = 0.145) and time point (*F* = 1.336, *p* = 0.262). Before stimulation, there was no significant difference in MEP amplitude between the real TUS condition (115.02 ± 21.7 μV) and the sham TUS condition (122.1 ± 24.23 μV, *t* = −2.012, *p* = 0.059). Moreover, the MEP amplitude after real TUS (132 ± 23.28 μV) was significantly higher than that obtained after the sham TUS (114.74 ± 25.5 μV, *t* = 3.193, *p* = 0.005, Figure 4). MEP amplitude significantly increased after stimulation compared to before stimulation in real TUS condition (*t* = 5.140, *p* < 0.001), however, there were no significant changes in MEP amplitude after stimulation on sham TUS condition (*t* = 1.111, *p* = 0.280, Figure 4).

### Comparison of discomfort

There were no significant differences between the discomfort scores associated with real TUS 5(5,5) and sham TUS 5(5,5) stimulation conditions (z = 1.414, *p* = 0.157).

## Discussion

Transcranial ultrasound stimulation (TUS) is a novel neuromodulation technique that has not yet been widely adopted in clinical practice (8, 13). In this study, TUS was used to stimulate the motor area of the brain in healthy adults, and it could enhance hand responsiveness, shorten the transmission time of MEPs, and enhance motor cortex excitability, indicating that low-frequency and low-intensity TUS has a positive effect on central excitability and hand function.

Some previous studies reported that TUS can regulate neuronal activity (8). The short-term neuronal activity involves inhibition or activation, while in the long term it may involve the reorganization of neural circuits (neuroplasticity), which could potentially improve brain function (7). A previous study has shown that transcranial-focused ultrasound increased cortical blood flow in the activated area of the motor cortex (27). Most researchers consider that the non-thermal effects of ultrasound play a role in the regulation of neuronal activity, including the opening of mechanically-gated ion channels and changes in membrane permeability caused by cavitation effects (13, 28). Due to the plasticity of the human cortex, low-intensity TUS can increase the excitability of the target brain circuit within a short time, suggesting that endogenous motor cortex activity can be enhanced by regulating excitability (29).

Our tapping score measurement included a comprehensive evaluation of speed and accuracy like the score calculation of archery. The results indicated that TUS could shorten the reaction time and improve the reaction ability of the hand by direct stimulation of the brain. Ichijo et al. found that low-intensity TUS improved the ability of mice affected by stroke to walk on a wire rope and navigate a maze. Few previous studies have focused on the effects of TUS on human behavior (30).

The results of this study show that the MEP latency was shortened, and the amplitude of the MEP was increased after TUS stimulation, suggesting that low-intensity TUS could enhance the excitability of the motor cortex. When TMS is applied to the primary motor cortex, corticospinal neurons are activated, and an MEP is generated in the target muscle (31). MEP latency reflects the conduction capacity of the corticospinal tract, and MEP amplitude can be used to determine changes in cortical excitability. In agreement with our results, Gibson et al. used diagnostic TUS applied to the cortical area for 2 min and found that the MEP amplitude was increased after stimulation (10). Using self-made ultrasound equipment, Legon et al. also showed that focused TUS (0.5 MHz, 6.16 W/cm^2^) can regulate central excitability (11). However, in their case, the MEP amplitude decreased after stimulation, suggesting inhibition of the motor cortex. These result differences may be related to the specific parameter setting of TUS. In the present study, we used non-focused ultrasound, while Legon et al. employed transcranial-focused ultrasound; also, the intensity we used was lower than that used in Legon's study. Nevertheless, both results provide evidence that TUS can regulate the excitability of the human motor cortex.

In the present study, VAS was used as a tool to monitor the discomfort of subjects, reflecting their subjective feelings about the treatment. Because the low-intensity TUS we used is commercially available and does not have any thermal effects, we expected the participants to not experience any noticeable sensation during the treatment session, and indeed there were no significant differences in the VAS scores between the two conditions. Liu et al. suggested based on animal experiments that the heat generated by low-intensity TUS is extremely weak, far below the temperature threshold capable of causing biological effects (32). This means that the local temperature in the target area may remain almost unchanged, and there is no danger of thermal damage to normal tissues (33). As a non-invasive form of brain stimulation, low-intensity TUS does not involve the opening of the blood–brain barrier, nor does it produce morphological changes in the brain (7), and therefore, it can be considered safe. During the course of our study, a single participant experienced mild dizziness after TUS treatment, which disappeared spontaneously within 5 min.

Our study has several limitations. First, we focused exclusively on the effect of a single TUS stimulus, even though it has a statistically significant difference in MEP latency between the real and sham conditions after stimulation, the changes in mean value were small. Further research is needed to determine the potential effects of a different stimulus schedule on excitability. Second, we observed the regulatory effect of TUS on the brains of healthy adults, and it is not clear whether the same changes would also occur in pathological conditions. Third, this study was restricted to the effects of TUS on hand motor function and central regulation, but the relevance of this effect to behavioral training remains unclear. Finally, the assessment method employed in this study is relatively limited, and therefore, it would be necessary to conduct further research on the impact of TUS on neural networks through methods involving neuroimaging in the future.

In conclusion, low-intensity TUS can improve hand motor function in healthy adults by shortening the latency of MEP and enhancing the excitability of the motor cortex.

## Data availability statement

The data that support the findings of this study are available from the corresponding author upon reasonable request.

## Ethics statement

The studies involving human participants were reviewed and approved by the Ethics Committee of Yue dong Hospital of the Third Affiliated Hospital of Sun Yat-sen University. The patients/participants provided their written informed consent to participate in this study.

## Author contributions

Z-MT and F-BH: conceptualization. M-FZ and Z-MT: methodology. W-ZC: assessment. M-FZ: TUS operator and writing—original draft preparation. Z-YP: formal analysis and investigation. Z-MT: writing—review and editing and funding acquisition. All authors contributed to the study conception and design. All authors contributed to the article and approved the submitted version.

## Funding

This work was supported by the National Natural Science Foundation of China (Grant Number: 81401872).

## Conflict of interest

The authors declare that the research was conducted in the absence of any commercial or financial relationships that could be construed as a potential conflict of interest.

## Publisher's note

All claims expressed in this article are solely those of the authors and do not necessarily represent those of their affiliated organizations, or those of the publisher, the editors and the reviewers. Any product that may be evaluated in this article, or claim that may be made by its manufacturer, is not guaranteed or endorsed by the publisher.
